# Overexpression of mitochondrial uncoupling protein 1 (UCP1) induces a hypoxic response in *Nicotiana tabacum* leaves

**DOI:** 10.1093/jxb/erv460

**Published:** 2015-10-22

**Authors:** Pedro Barreto, Vagner Okura, Izabella A. Pena, Renato Maia, Ivan G. Maia, Paulo Arruda

**Affiliations:** ^1^Centro de Biologia Molecular e Engenharia Genética, Universidade Estadual de Campinas (UNICAMP),13083-875 Campinas, SP, Brazil; ^2^Departamento de Genética e Evolução, Instituto de Biologia, Universidade Estadual de Campinas (UNICAMP), 13083-875 Campinas, SP, Brazil; ^3^Departamento de Genética, Instituto de Biociências, UNESP, 18618-970 Botucatu, SP, Brazil

**Keywords:** Carbon fixation, hypoxic stress, oxidative stress, photosynthesis, stress tolerance, UCP1.

## Abstract

Mitochondrial uncoupling protein 1 (UCP1) decreases reactive oxygen species production under stress conditions by uncoupling the electrochemical gradient from ATP synthesis. This study combined transcriptome profiling with experimentally induced hypoxia to mechanistically dissect the impact of *Arabidopsis thaliana* UCP1 (AtUCP1) overexpression in tobacco. Transcriptomic analysis of AtUCP1-overexpressing (P07) and wild-type (WT) plants was carried out using RNA sequencing. Metabolite and carbohydrate profiling of hypoxia-treated plants was performed using ^1^H-nuclear magnetic resonance spectroscopy and high-performance anion-exchange chromatography with pulsed amperometric detection. The transcriptome of P07 plants revealed a broad induction of stress-responsive genes that were not strictly related to the mitochondrial antioxidant machinery, suggesting that overexpression of AtUCP1 imposes a strong stress response within the cell. In addition, transcripts that mapped into carbon fixation and energy expenditure pathways were broadly altered. It was found that metabolite markers of hypoxic adaptation, such as alanine and tricarboxylic acid intermediates, accumulated in P07 plants under control conditions at similar rates to WT plants under hypoxia. These findings indicate that constitutive overexpression of AtUCP1 induces a hypoxic response. The metabolites that accumulated in P07 plants are believed to be important in signalling for an improvement in carbon assimilation and induction of a hypoxic response. Under these conditions, mitochondrial ATP production is less necessary and fermentative glycolysis becomes critical to meet cell energy demands. In this scenario, the more flexible energy metabolism along with an intrinsically activated hypoxic response make these plants better adapted to face several biotic and abiotic stresses.

## Highlight

Evidence was found linking alterations in carbon fixation and mitochondrial metabolism that lead to increased stress tolerance in UCP1-overexpressing plants.

## Introduction

Mitochondrial uncoupling proteins (UCPs) comprise a family of nuclear-encoded proteins that are localized to the mitochondrial inner membrane (Krauss *et al.*, 2005; Vercesi *et al.*, 2006). Although the biochemical mechanisms and relevance of UCPs are not completely understood, it is known that its activation results in the re-entry of H^+^ from the intermembrane space back into the mitochondrial matrix, thus reducing oxidative phosphorylation (OXPHOS) efficiency (Figueira *et al.*, 2013). In brown adipose tissue of animals, UCP1 has a thermogenic role, dissipating the electrochemical gradient as heat (Nicholls and Rial, 1999). The identification of UCP1 homologues in humans (Krauss *et al.*, 2005) and orthologues in several eukaryotic organisms (Vercesi *et al.*, 2006) suggests that other physiological functions exist for these proteins in addition to thermogenesis. UCPs may serve as escape valves, decreasing the proton motive force and preventing the production of reactive oxygen species (ROS) under unfavourable conditions or when the cellular demands for ATP are low (Krauss *et al.*, 2005; Vercesi *et al.*, 2006; Mailloux *et al.*, 2011, 2012; Figueira *et al.*, 2013).

In plants, members of the UCP family have been associated with several physiological roles, including thermogenesis regulation (Ito-Inaba *et al.*, 2008), abiotic stress responses (Brandalise *et al.*, 2003; Smith *et al.*, 2004; Begcy *et al.*, 2011; Figueira and Arruda, 2011; Chen *et al.*, 2013; Barreto *et al.*, 2014), and climacteric increases in respiration (Jezek *et al.*, 2001). The genes encoding UCP1 and other UCP-related proteins are expressed in a time- and tissue-dependent manner and are upregulated by cold stress in plants (Maia *et al.*, 1998; Borecký *et al.*, 2006; Vercesi *et al.*, 2006). The overexpression of UCPs protects plants from biotic (Chen *et al.*, 2013) and abiotic stresses, including oxidation, high salt, drought, and heat (Brandalise *et al.*, 2003; Smith *et al.*, 2004; Begcy *et al.*, 2011; Figueira and Arruda, 2011; Chen *et al.*, 2013). In addition, UCP-overexpressing plants exhibit increased carbon assimilation and stomatal conductance under both control and stressful conditions (Begcy *et al.*, 2011). The phenotype of plants overexpressing UCPs have been explained in terms of decreased ROS production by the mitochondrial electron transport chain (Brandalise *et al.*, 2003; Begcy *et al.*, 2011; Chen *et al.*, 2013), which benefits not only adaptation of the plant to biotic and abiotic stresses but also the plant’s capacity to assimilate carbon under control conditions.

Nevertheless, it was recently shown that overexpression of the *Arabidopsis* mitochondrial UCP1 (AtUCP1) in tobacco has a more widespread effect rather than simply decreasing ROS production (Barreto *et al.*, 2014). The constitutive uncoupling of respiration results in increased oxygen consumption in isolated mitochondria, decreased cellular ATP concentration, altered mitochondrial morphology, and triggers a retrograde response. As a result, mitochondrial biogenesis increases and an array of both nuclear- and mitochondria-encoded genes are induced, including many that encode components of the stress response. It has been concluded that UCP1 overexpression might be triggering a metabolic dysfunction and enhancing a strong stress response, which results in plants being better adapted to a number of biotic and abiotic stresses (Barreto *et al.*, 2014). However, the reason why uncoupling of protein overexpression triggers this broad stress response remains poorly understood (Barreto *et al.*, 2014), as well as how it relates to previous observations (Brandalise *et al.*, 2003; Smith *et al.*, 2004; Begcy *et al.*, 2011; Chen *et al.*, 2013).

Here, transcriptome and metabolite profiling were combined, along with experimentally induced hypoxic stress, to determine the mechanism underlying the impact of overexpressing the gene encoding AtUCP1in tobacco plants. Taken together, the data support the notion that UCP1 overexpression induces a hypoxic stress that disrupts cellular energy homeostasis and triggers a reconfiguration of metabolism.

## Materials and methods

### Plant material and growth conditions

Tobacco (*Nicotiana tabacum*) ecotype SR1 overexpressing AtUCP1 was obtained as described previously (Brandalise *et al.*, 2003). In this study, the transgenic line P07 was used, which constitutively expressed high amounts of AtUCP1 (Brandalise *et al.*, 2003; Barreto *et al.*, 2014). P07 and wild-type (WT) seeds were planted in pots containing soil:vermiculite (1:1) mixture. Plants were grown in a growth chamber with a photoperiod regime of 16h of light (100 µE m^–2^ s^–1^) and 8h of darkness. For the molecular analysis, leaves from 12-week-old plants were harvested, immediately frozen in liquid nitrogen, and stored at –80 °C. Four biological replicates were sampled for each genotype.

For hypoxic treatments, 4-week-old P07 and WT plants were placed in a sealed glass chamber with a constant flow of an air mixture composed of 5% O_2_, 0.04% CO_2,_ and 94.96% N_2_ for 48h. The aboveground parts of the plants subjected to hypoxia were collected at the end of the light period (0, 24, and 48h), during the dark period (2h), and at the end of the dark period (8h). At the end of the treatment, plants were placed in a normal atmosphere for 8h to recover. Control plants were maintained at normoxia throughout the experiment and collected at the same time as the hypoxia-treated plants to serve as controls. Samples were collected, immediately frozen in liquid nitrogen, and stored at –80 °C. Four biological replicates, each comprising a pool of three plants, were sampled for each genotype.

### Preparation of a cDNA library, transcriptome sequencing, mapping, and annotation

A schematic representation of the pipeline used for the transcriptome assembly, mapping, and annotation described in this section is also shown in Supplementary Fig. S1 (available at *JXB* online). Single-end RNA-sequencing libraries were prepared as described previously (Barreto *et al.*, 2014). The transcriptome profiles of P07 tobacco plants overexpressing AtUCP1 and WT counterparts were assessed using the leaves of 12-week-old plants. The libraries were sequenced in four lanes using the Illumina HiSeq 2000 sequencing system for 75 cycles. A total of 42.7 Gb of sequence data was generated for eight libraries with a minimum of 27.9 million reads and a maximum of 113.2 million reads for each library (Supplementary Table S1A, available at *JXB* online). The Illumina reads were filtered to remove adapters and low-quality reads (reads with <70% of the bases possessing quality scores of ≥Q20) using AdapterRemoval (Lindgreen, 2012), the FASTX-Toolkit (http://hannonlab.cshl.edu/fastx_toolkit), and Perl scripts. The resulting 569 million high-quality reads were normalized using diginorm software (https://github.com/ged-lab/2012-paper-diginorm), resulting in 49.5 million normalized reads. These were assembled into 271 750 contigs with an average length of 1163bp (Supplementary Table S1B). Because the *N. tabacum* genome has not yet been completely sequenced and the scaffolds of *Nicotiana benthamiana* are estimated to cover only 79% of its genome, the complete genome sequence of *Solanum lycopersicum* (Tomato Genome Consortium, 2012) was used, which is a close relative of *N. tabacum*, to map and annotate the expressed contigs. To identify the protein-coding genes, all of the contigs were used as queries in BLASTn and BLASTx searches against both the non-redundant set of tomato coding sequences and the predicted protein sequences. Of the 271 750 contigs, 134 752 mapped to the tomato genome, resulting in annotation of 20 045 distinct orthologues for the tomato coding sequences models. The 569 million high-quality reads were mapped to the 271 750 assembled contigs, and gene expression amounts were expressed as reads per kb per million (RPKM) values, which ranged from 1×10^–3^ to over 2×10^4^. An RPKM cut-off value of 0.1 was set to indicate contig expression (Supplementary Table S1B). The resulting 9644 annotated transcripts were mapped onto the *Arabidopsis thaliana* transcriptome using Blast2GO (Conesa *et al.*, 2005) and biological pathways using MapMan (Thimm *et al.*, 2004). The sequence data are available in the Sequence Read Archive, which is accessible through NCBI BioProject ID PRJNA211804 under experiment IDSUB287723.

### Functional annotation of the differentially expressed genes (DEGs)

Functional annotation was carried out only for DEGs with RPKM values that had a ≥1.5-fold change and were significantly different between WT and P07. The DEGs was determined by performing Kal’s *Z*-test using a *P* value cut-off of 0.05 after adjusting for the false discovery rate (FDR), set at 5%. A complete list of the genes determined as upregulated and downregulated in transgenic plants overexpressing AtUCP1 compared with its WT counterpart is available in Supplementary Table S2 (available at *JXB* online). Gene function annotations were based on existing annotations for the tomato genome and by BLASTp searches against the UniProt database (UniProt Consortium, 2014). The resulting protein dataset was mapped against the Eukaryotic Clusters of Orthologous Groups (COG) (Tatusov *et al.*, 2003) database by BLASTp, and COG functional categories were assigned only if the two best hits had the same COG function. The Blast2GO software (Conesa *et al.*, 2005) was used to map the DEGs to the *Arabidopsis* proteome. Gene Ontology (GO) (Gene Ontology Consortium, 2000) categories and Kyoto Encyclopedia of Genes and Genomes (KEGG) (Kanehisa and Goto, 2000) pathways were then assigned to the transcripts using the Blast2GO tool. For GO term enrichment analysis using Fischer’s exact test, all DEGs were mapped to GO terms and significantly enriched ontologies were searched for compared with the whole transcriptome background. Transcription factors (TFs) were mapped by BLASTp searches against the Plant Transcription Factor Database (PlnTFDB 3.0) (Perez-Rodriguez *et al.*, 2009), and Perl scripts were used to identify only the bidirectional best hits. Annotations of TFs were assigned to the corresponding representative tobacco contiguous sequence (contig). TF expression profiles and co-expression analyses were carried out using Genevestigator software (Hruz *et al.*, 2008). The over-representation analysis performed for the genes co-expressed with the selected TFs was carried out using MapMan (Thimm *et al.*, 2004). The top nine up- and downregulated TFs between WT and P07 plants were identified, as well as selected publicly available experimental datasets where the expression of these TFs was significantly altered. The genes that were co-expressed with each of the TFs were then analysed, and those that were positively or negatively correlated with individual TFs were selected. These genes were grouped into those that were positively and negatively correlated with the upregulated and downregulated TFs. The over-representation analysis was performed individually for each of these groups using the whole *Arabidopsis* genome as template. A schematic representation of the pipeline for the TF co-expression analysis is shown in Supplementary Fig. S2 (available at *JXB* online).

### Quantitative reverse transcription (qRT)-PCR analysis

Total RNA that was isolated from WT and P07 plants was used for first-strand cDNA synthesis using a Revertaid First Strand cDNA Synsthesis kit (Fermentas, Vilnius, Lithuania) according to the manufacturer’s protocol. Real-time PCR was performed using the ABI PRISM 7500 system (Applied Biosystems, Foster City, CA, USA) with SYBR Green dye (Applied Biosystems). The reactions were performed at least in triplicate with four biological replicates, and the results were expressed relative to the expression levels of the *N. tabacum Actin1* gene in each sample using the 2^–ΔΔ*C*^_T_ method. All values are presented as fold changes of P07 relative to WT. Student’s *t*-test was performed to determine significant changes (*P*<0.1).

Thirteen contigs that were unaltered or were up- or downregulated in P07 compared with WT plants were randomly selected and used for experimental expression validation of the transcriptomic data (Supplementary Table S3A, available at *JXB* online). The fold-change values that were obtained from the transcriptome assembly showed a high correlation with the qRT-PCR data (Supplementary Fig. S3A, available at *JXB* online) with an *R* ^2^ value of 0.9693 (Supplementary Fig. S3B).

A specific subset of genes was selected to evaluate the performance of P07 and WT plants subjected to hypoxic conditions (Supplementary Table S3B).

### Metabolite profiling and carbohydrates analysis

P07 and WT plants submitted to hypoxic treatment for 8h were used for metabolic profiling by ^1^H-nuclear magnetic resonance (NMR) spectroscopy. Metabolites were isolated as described elsewhere (Häusler *et al.*, 2000; Kaiser *et al.*, 2008) with minor modifications. A total of 100mg of frozen material was pulverized in liquid nitrogen. Samples were kept in contact with 1ml of extraction solution containing chloroform:methanol:water (2:4:1) for 30min in ice, and vigorously homogenized for an additional 15min. After the addition of 1ml of water, samples were centrifuged at 12 200*g* for 5min for phase separation. The methanol:water phase were collected and vacuum dried for 8h. Four biological replicates for control and hypoxia-treated plants were used for metabolite isolation procedures.

After drying the samples, the remaining solid phase was rehydrated in 600 µl of D_2_O-containing phosphate buffer (0.1M, pH 7.4) and 0.5mM of 3-(trimethylsilyl)propanoic acid for internal reference. Samples were added to a 5mm NMR tube for immediate acquisition. ^1^H-NMR spectra of the samples were acquired using an Agilent Inova NMR spectrometer (Agilent Technologies, Santa Clara, CA, USA) equipped with a 5mm triple resonance cold probe and operating at a ^1^H resonance frequency of 600 MHz and constant temperature of 25 °C. A total of 128 free induction decays were collected with 32 K data points over a spectral width of 16 ppm. A 1.5 s relaxation delay was incorporated between free induction decays, during which a continual water pre-saturation radiofrequency field was applied. Spectral phase and baseline corrections, as well as the identification and quantification of the metabolites present in the samples, were performed using the Chenomx NMR Suite 7.6 software (Chenomx, Edmonton, AB, Canada).

Soluble sugars and starch were extracted and quantified as described previously (Stitt *et al.*, 1989; Hendriks *et al.*, 2003). Total soluble sugars and starch were extracted three times with 80% (v/v) ethanol at 80 °C for 20min. The supernatants were pooled, dried using a centrifugal vacuum concentrator, resuspended in pure water, and filtered. Sugars were analysed by high-performance anion-exchange chromatography with pulsed amperometric detection (HPAEC-PAD; ICS 3000, Thermo Scientific Dionex) on a CarboPac PA-1 4×250mm column set (Thermo Scientific Dionex) using a gradient of eluent A (water) and eluent B (200mM NaOH), and a flow rate 0.8ml min^–1^ over 22min as follows: 0–12min, 50% B/50% A; 12.1–17min, 100% B; and 17.1–22min, 50% B/50% A. Glucose, fructose, and sucrose were identified and quantified by comparison with original standards using Chromeleon software (version 6.8; Thermo Scientific Dionex). Starch was determined in the insoluble fraction after ethanolic extraction. The starch was solubilized in 0.1M NaOH for 30min at 95 °C. After neutralization, the starch was digested to glucose by the addition of amyloglucosidase and α-amylase at 37 °C overnight. To assess the starch level in the samples, the glucose content was assayed enzymatically by coupling to the reduction of NADP^+^ to NADPH in a microplate reader.

### Chlorophyll fluorescence and chlorophyll content analysis

Measurements were performed on illuminated samples and after a 20min period of dark acclimation using an imaging pulse amplitude modulated fluorimeter (Heinz Walz, Effeltrich, Germany). All parameters related to fluorescence quenching were calculated using four notable values obtained from two standard measurement procedures on dark-adapted and light-adapted plants according to the manufacturer’s protocol. A first fluorescence measurement on light-adapted plants under an actinic light source followed by a second measurement after a short pulse of saturated light gave, respectively, the minimum (F′) and the maximum (Fm′) fluorescence emission from leaf samples under standard illumination conditions, which allowed quantification of the effective photochemical quantum yield [Y(II)] (Fq′/Fm′]. After such measurements, leaf clips were used to prevent leaves being exposed to light for 20min in order to completely oxidize the reaction centres of thylakoid membranes (dark-adapted plants). This allowed the measurement of minimum fluorescence emission after exposure to non-actinic light (Fo), and the measurement of maximum fluorescence emission after a saturated light pulse upon dark-adapted leaf samples (Fm), which allowed calculation of the maximum photochemical quantum yield of photosystem II (PSII) (Fv/Fm) and PSII maximum efficiency (Genty Factor) (Fv′/Fm′). The coefficient of photochemical fluorescence quenching (qP) was also quantified for both genotypes. Leaf samples were collected immediately after chlorophyll fluorescence measurements and stored at –80 °C for chlorophyll quantification.

Chlorophyll extraction and quantification were performed as described previously (Ni *et al.*, 2009) with minor modifications. Leaves (30mg) ground in liquid nitrogen were incubated for 30min in 1ml of 80% acetone. The homogenate was centrifuged at 4000*g* at 4 °C for 15min and the supernatant was collected. The incubation with 1ml of 80% acetone was repeated and the supernatants were combined. All the steps of chlorophyll extraction were performed in the dark to avoid degradation. Extracts were diluted five times and used to determine the level of chlorophyll A (663nm) and chlorophyll B (645nm) in a spectrophotometer.

## Results

### Transcriptome pathway mapping

**Fig. 1.**
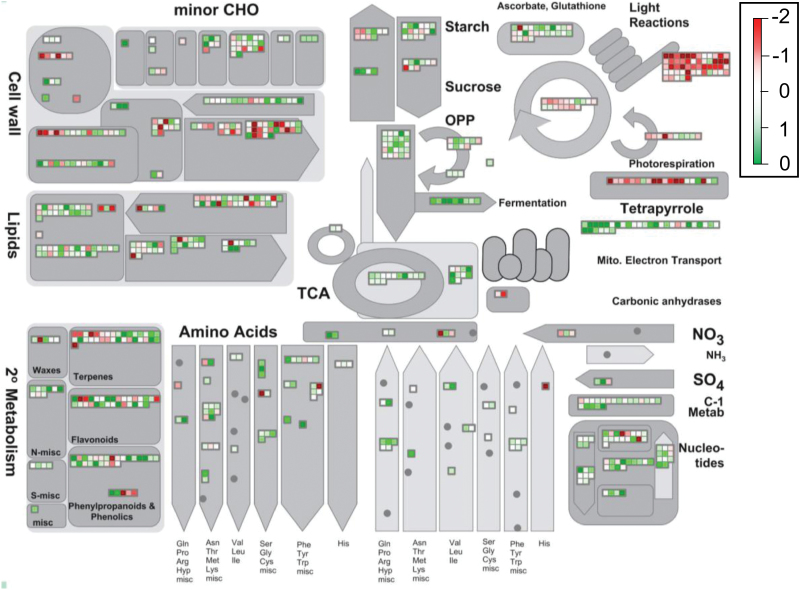
Mapman metabolic overview of the UCP1-overexpressor transcriptome. The 9822 transcripts declared as expressed (RPKM ≥0.1) were mapped into *A. thaliana* major metabolic pathways. Processes related to carbon fixation pathways appear to be repressed in UCP1 overexpressors. In contrast, transcripts related to energy expenditure processes seem to be induced in transgenic plants. The log_2_ ratio of the transgenic to the WT for individual genes was plotted onto boxes. Green boxes indicate upregulated genes and red boxes indicate downregulated genes.

As a starting point to observe the altered metabolic pathways due to UCP1 overexpression, a total of 9822 transcripts, determined as expressed in both WT and P07 plants, were mapped into *A. thaliana* transcriptome and biological pathways. The metabolic overview of the transcriptome revealed alterations in the major metabolic processes inside a plant cell (Fig. 1). The transcripts that mapped into carbon fixation processes, such as ‘photosynthesis light reactions’, ‘calvin cycle’, ‘tetrapyrrole synthesis’, and ‘photorespiration’ seemed to be broadly downregulated in transgenic plants (Fig. 1). It is important to highlight that these plants had increased carbon assimilation and stomatal conductance when compared with WT (Begcy *et al.*, 2011). In the other direction, transcripts that mapped into the pathways related to energy expenditure, which included ‘sugar degradation’, ‘glycolysis’, ‘fermentation’, ‘oxidative pentose phosphate’, ‘tricarboxylic acid cycle (TCA)’, and ‘mitochondrial electron transport chain’, were mostly upregulated.

### Functional annotation and enrichment analysis of DEGs

To verify whether the transcriptome alteration overview was significant, a more detailed functional annotation was carried out for the DEGs only. The DEGs that were annotated by matching with the tomato coding sequences were used as queries in a BlastP search against the UNIPROT database. To further evaluate the effectiveness of the annotations, the annotated sequences were searched for gene transcripts with COG classifications. This enabled classification of the 1679 upregulated and 550 downregulated transcripts into 25 functional categories (Supplementary Fig. S4, available at *JXB* online). GO assignments were also applied to classify the functions of the DEGs using Blast2GO (Supplementary Table S4, available at *JXB* online).

Fisher’s exact test enrichment analysis (Auer and Doerge, 2010) was used to identify the most over-represented ontologies and pathways of the up- and downregulated groups of gene transcripts. Among the upregulated transcripts, the ontologies ‘response to stress’, ‘transcription factor activity’, and ‘mitochondrion’ were among the most highly represented (Table 1). Consistent with our previous observations that there was a broad activation of stress-responsive genes (Barreto *et al.*, 2014), this category was over-represented in the upregulated group of genes. To visualize the expression pattern of the stress-responsive genes and which category they belonged to, the transcriptome mapping into MapMan categories was analysed and related to stress (Supplementary Fig. S5, available at *JXB* online). It was found that both biotic and abiotic stress-responsive genes were induced, consistent with the observation that tomato plants overexpressing an UCP are more tolerant to pathogen attack (Chen *et al.*, 2013). Interestingly, this broad induction of stress-responsive genes was not limited to the mitochondrial antioxidant machinery.

**Table 1.** GO enrichment analysis for the DEGs in UCP1-overexpressing plants

Upregulated		Downregulated	
GO term	*P* value	GO term	*P* value
Nucleic acid binding transcription factor activity	6.48E–04	PSII associated light-harvesting complex II	8.53E–05
Organellar ribosome	6.42E–04	Thylakoid light-harvesting complex	8.53E–05
Signal transduction	6.34E–04	Response to red light	4.05E–05
Protein targeting to mitochondrion	8.02E–05	Nutrient reservoir activity	1.22E–05
Mitochondrion	6.46E–05	Chlorophyll biosynthetic process	7.14E–08
Response to ethylene stimulus	5.18E–05	Photosystem I	4.02E–10
Response to stress	1.06E–05	Tetrapyrrole binding	8.73E–12
Fermentation	3.02E–07	Photosystem	5.25E–12
Purine nucleotide binding	2.72E–07	Photosynthesis	2.88E–13
Mitochondrial electron transport	1.62E–07	Chlorophyll binding	1.59E–13
Protein kinase activity	8.50E–08	Photosynthesis, light reaction	3.10E–14
Negative regulation of cell death	4.60E–08	Photosynthetic membrane	1.19E–14
ADP binding	8.56E–10	Light-harvesting complex	6.37E–16
Defence response	9.56E–12	Thylakoid	1.27E–17

The KEGG database was also used to map the DEGs onto the biological pathways that were altered in P07 compared with WT plants. Consistent with the GO analysis, ‘purine metabolism’ (38 genes) was the most representative pathway, together with ‘glycolysis’, ‘tricarboxylic acid cycle (TCA)’, ‘OXPHOS’ and ‘starch and sucrose metabolism’ (Supplementary Table S5, available at *JXB* online). Plant cells use ribose 5-phosphate from oxidative pentose phosphate for *de novo* nucleotide synthesis. Interestingly, the oxidative pentose phosphate pathway seems to be induced (Fig. 1) and purine metabolism over-represented in tobacco plants overexpressing AtUCP1. The transcript related to guanylate kinase, which converts GMP into GTP is upregulated 3-fold in transgenic plants (Supplementary Fig. S6, available at *JXB* online). Purine nucleotides, especially GTP, are common inhibitors of UCP activity (Smith *et al.*, 2004; Figueira *et al.*, 2013). The alterations observed regarding sucrose metabolism were primarily mapped to sucrose synthesis from UDP-glucose by sucrose phosphate synthase (ATSPS3F and ATSPS4F) and breakdown by invertases (Supplementary Fig. S7A, available at *JXB* online). Our data also showed that starch breakdown into glucose and maltose was induced in UCP1 overexpressors (Supplementary Fig. S7B).

Interestingly, several ontologies related to chloroplast metabolism were among the enriched terms of the downregulated genes in the P07 plants (Table 1). For this reason, the expression of genes encoding chloroplast proteins was further analysed in an attempt to elucidate the impact of UCP1 overexpression on metabolism in this organelle.

### UCP1 overexpression affects chloroplast metabolism

**Fig. 2.**
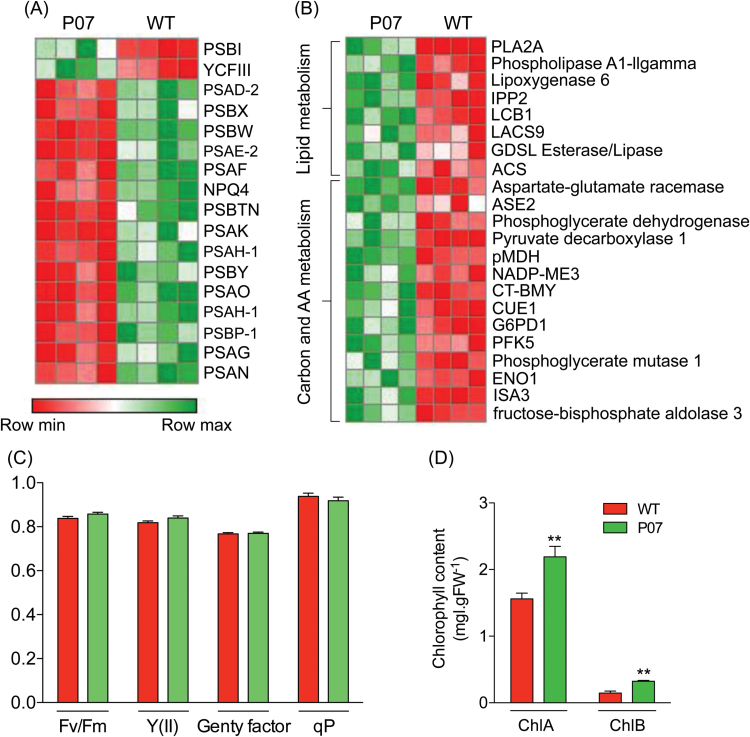
Overexpression of AtUCP1 alters chloroplast metabolism. (A) Genes targeted to PSI and PSII reaction centres are downregulated in transgenic plants. (B) Carbon, amino acid, and lipid metabolism were the most represented categories of the upregulated genes encoding chloroplast proteins. Hypoxic pathways represented by PDC and ACS were upregulated in the AtUCP1 transgenic plants. The expression scale varies from dark red (downregulation) to dark green (upregulation). (C) There was no alteration in the photosynthesis parameters between AtUCP1 transgenic plants compared with the WT when plants were grown under normal conditions. (D) AtUCP1-overexpressing plants showed increased in chlorophyll A and B content compared with the WT when plants were grown under normal conditions. Bars represent mean ±SEM. **, Significantly from the WT (*P*<0.1).

The balance between energy supply and demand depends largely on a tightly regulated signalling network that coordinates photosynthesis, photorespiration, and respiration, three of the most critical processes to plant life (Suzuki *et al.*, 2012). Thus, it is reasonable to expect that the metabolic alterations promoted by AtUCP1 overexpression would also affect the expression of genes involved in chloroplast metabolism. Although many gene transcripts encoding chloroplast-targeted proteins were induced in P07 plants, only two of these encoded subunits of the photosynthesis reaction centres (Fig. 2A and Supplementary Table S6, available at *JXB* online). Both of these have unknown functions and are encoded by the chloroplast genome. Conversely, several transcripts encoding components of the PSI and PSII complexes were downregulated 1.8- to 2.4-fold in P07 plants. Despite the decreased PSI and PSII transcript levels, chlorophyll fluorescence measurements revealed no significant alterations between the P07 and WT plants for any of the quantified parameters (Fig. 2C) and the chlorophyll content displayed an opposite directional change by being elevated in the P07 plants compared with the WT plants (Fig. 2D).

To elucidate the impact of the alterations on chloroplast gene expression, further annotation analyses were carried out on the chloroplast genes that were upregulated in the P07 plants (Fig. 2B and Supplementary Table S6). This revealed that most of the highly upregulated chloroplast-related gene transcripts were involved in carbon, lipid, and amino acid metabolism. Lipid biosynthesis can be achieved by two distinct pathways in plants, one is the plastid pyruvate-dehydrogenase complex (pPDHC), which is responsible for the majority of fatty acid biosynthesis (Schwender *et al.*, 2006) and another is the plastid acetyl-CoA synthase (ACS) (Lin and Oliver, 2008). Therefore, both of these pathways are involved in providing acetyl-CoA for lipid biosynthesis inside chloroplasts. The expression of genes encoding components of the pPDHC complex did not change in the P07 plants compared with the WT. The lipid biosynthesis pathway that uses ACS bypasses the PDHC complex, converting the toxic by-products of fermentative glycolysis into fatty acids (Lin and Oliver, 2008). The transcript encoding ACS was strongly upregulated in the P07 plants (Fig. 2B and Supplementary Table S6).

### Transcriptome assessment of hypoxic stress imposed by AtUCP1 overexpression

**Fig. 3.**
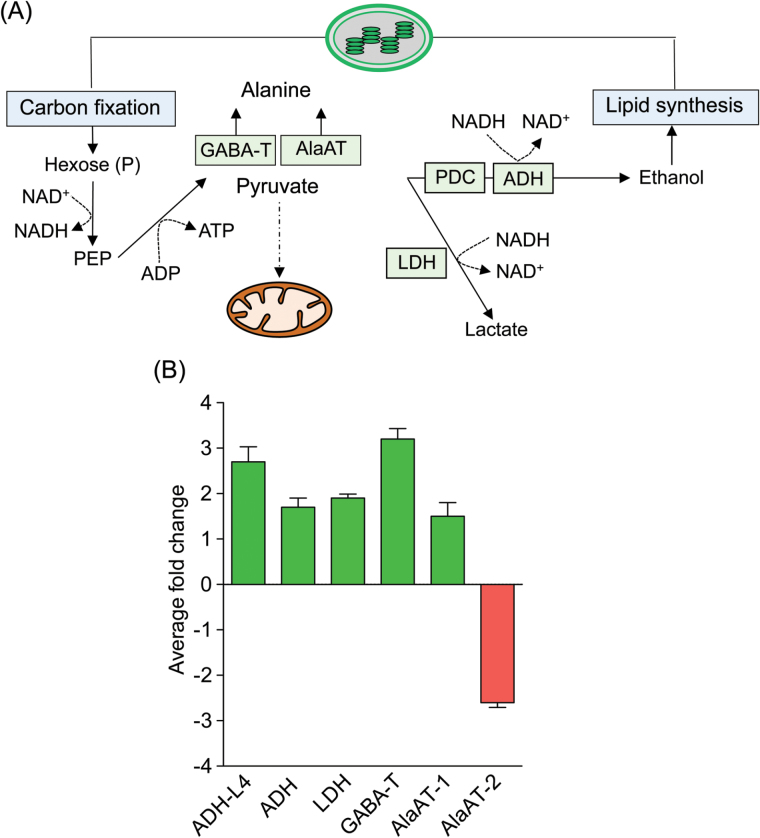
Metabolic consequences of hypoxia. (A) Under hypoxia, pyruvate is converted to alanine by the action of *γ*-aminobutyric acid transaminase (GABA-T) or alanine aminotransferase (AlaAT) in order to prevent its flow to mitochondria. Pyruvate is used for NAD^+^ regeneration in the cytosol by the action of PDC, ADH, and LDH. (B) Transcriptomic analysis showed increased transcript levels of markers of hypoxic adaptation in the AtUCP1 transgenic plants compared with the WT plants. PEP, phosphoenolpyruvate.

It was decided to investigate whether UCP1 overexpression would affect transcripts that are induced upon hypoxia. Mitochondria consume 80–90% of oxygen in the cells to support OXPHOS, which is the primary metabolic pathway for ATP production. Therefore, hypoxia will impair this pathway and impose metabolic adaptations to satisfy cellular energy demands. As oxygen becomes limiting, the OXPHOS process decreases ATP production and the electron transport chain decreases NADH oxidation and NAD^+^ regeneration, which in turn slows down the TCA. In such a situation, pyruvate should be used to regenerate NAD^+^ through fermentative glycolysis (Rocha *et al.*, 2010). This reaction can be performed either by lactate dehydrogenase (LDH) or by the subsequent reactions catalysed by pyruvate decarboxylase (PDC) and alcohol dehydrogenase (ADH) (Fig. 3A). Both the transcripts were upregulated in the P07 plants compared with the WT (Fig. 3B). NAD^+^ regeneration occurs through glycolysis, which in this situation would be the major source of cellular ATP. Transgenic plants were clearly inducing the initial steps of glycolysis and fermentative pathways (Supplementary Fig. S8, available at *JXB* online). Anaerobic glycolysis consumes pyruvate at a slower rate than OXPHOS and therefore pyruvate tends to accumulate within the cell. To prevent flow to the mitochondria and thereby control the rate of respiratory oxygen consumption, pyruvate can be converted into alanine via alanine aminotransferase (AlaAT) or *γ*-aminobutyric acid transaminase (GABA-T). In this study, the P07 plants may have produced alanine through the latter pathway, as GABA-T transcript levels were increased by 3.1-fold in P07 plants, while AlaAT-1 was unaltered and AlaAT-2 was decreased by 2.8-fold (Fig. 3B). Interestingly, these plants seemed to be removing TCA intermediates from the mitochondria using malate dehydrogenase and ATP citrate lyase in order to provide phosphoenolpyruvate for glycolysis and decrease TCA and OXPHOS reactions (Supplementary Fig. S8).

**Fig. 4.**
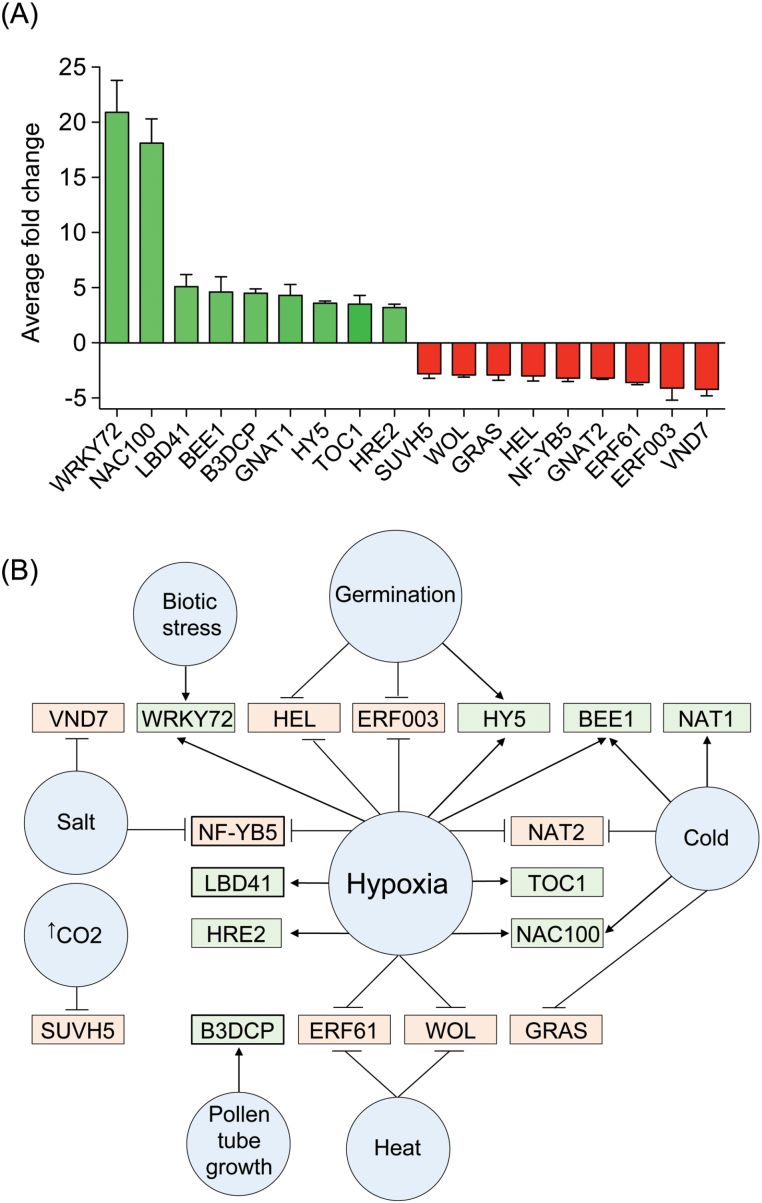
Characterization of differentially expressed TFs in AtUCP1 transgenic plants. The 18 most differentially expressed TFs were select for further evaluation using Genevestigator. (A) The most upregulated (green) or downregulated (red) TFs were selected. TFs that were downregulated in AtUCP1-overexpressing plants are shown in red boxes, and those that were upregulated are shown in green boxes. Bars represent means±SEM. The representative perturbations significantly affected each transcription factor gene expression by at least 2-fold (*P*<0.01). (B) The selected TFs were categorized accordingly to perturbations (blue circles) that impacted on their expression according to Genevestigator.

The expression profile of TFs potentially mediating the observed metabolic alterations was also analysed. Using the DEGs as queries in a search against the PlnTFDB 3.0 database allowed identification of 283 TFs that were differentially expressed, most of which have still not been functionally characterized in plants. The most representative DEG TF family was AP2-EREBP, which accounted for 21 members. The nine TFs with the highest fold changes were analysed, and the same number with the greatest decreases using Genevestigator (Fig. 4A). The hypoxia responsible element-2 (HRE2) was among one of the most highly induced TFs and has already been implicated in regulation of the hypoxia response (Licausi *et al.*, 2010; Gibbs *et al.*, 2011). It was also found that 13 of the differentially expressed TFs in P07 compared with WT plants were induced or repressed by hypoxic conditions (Fig. 4B). A group of genes that were co-expressed with these TFs was further selected (Supplementary Fig. S2). Gene enrichment analysis for the transcripts co-expressed with the up- and downregulated TFs showed that energy metabolism, including fermentation pathways, and stress responses were the most over-represented categories influenced by these regulators (Supplementary Table S7, available at *JXB* online).

### Gene expression and metabolite profiling of UCP1 overexpressors under hypoxic conditions

**Fig. 5.**
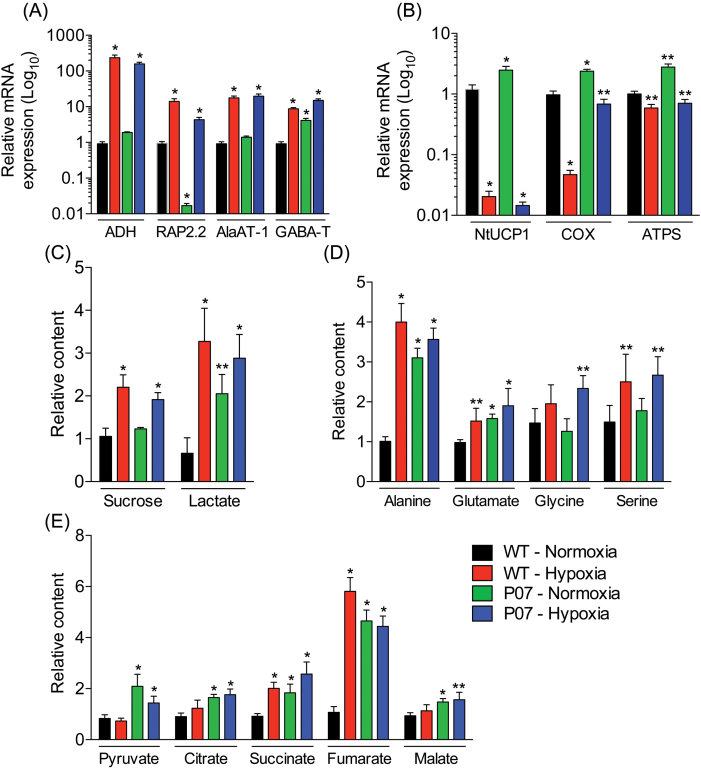
Metabolite and transcript profiles of AtUCP1-overexpressing and WT plants grown for 8h under hypoxia compared with plants grown in normoxia. (A, B) Transcript expression profiles of markers of hypoxic adaptation (A) and mitochondrial energy metabolism (B). (C, D) Metabolite profiles for carbohydrates and cytosolic metabolites (C), amino acids (D), and TCA intermediates (E). Bars represent means±SEM. **P<0.05; *P<0.1 (significantly different from the WT under normoxia).

To determine whether AtUCP1 overexpression indeed provoked a hypoxic response, WT and P07 plants were subjected to a 5% oxygen atmosphere and analysed the resulting hypoxia-responsive gene transcripts and metabolite profiles. The expression of genes (*ADH*, *AlaAT-1*, *GABA-T*, and the ethylene-responsive transcription factor *RAP2.2*), metabolites, and carbohydrates that are already known to be involved in hypoxic adaptation were also profiled. The transcript levels of ADH were increased in both P07 and WT plants grown for 2h under hypoxia compared with plants grown under normoxic conditions (Supplementary Fig. S9, available at *JXB* online). After 8h under hypoxia, *ADH* transcripts were increased by 200-fold in both P07 and WT plants (Fig. 5A). Consistent with the observed transcriptomic data, *ADH* transcripts were found to be 2-fold higher in P07 plants compared with the WT plants when both were grown under normoxic conditions for 8h. The TF RAP2.2 is known to be induced at early stages in plants grown under hypoxia (Hinz *et al.*, 2010). Under hypoxic conditions, the *RAP2.2* transcript level was increased 8- and 11-fold in P07 and WT plants, respectively, compared with plants grown in normoxia (Fig. 5A). Interestingly, *RAP2.2* transcription was strongly repressed in P07 compared with WT plants under the normal oxygen conditions. Following the 8h hypoxia treatment, transcript levels of both *AlaAT-1* and *GABA-T* were increased 11- and 8-fold in P07 and WT plants, respectively (Fig. 5A). In keeping with the transcriptomic data, *GABA-T* but not *AlaAT-1* transcripts were detected at 5-fold higher levels in P07 plants compared with WT plants under normoxic conditions (Fig. 5A).The transcripts of the endogenous tobacco *NtUCP1* and nuclear-encoded mitochondrial proteins involved in OXPHOS were also evaluated (Fig. 5B). The levels of the endogenous *NtUCP1* transcript were 2-fold higher in P07 compared with WT plants under normoxia, similar to the findings above. However, plants grown for 8h under hypoxic conditions contained *NtUCP1* transcripts that were reduced by 100- and 50-fold in P07 and WT plants, respectively. Interestingly, the transcript levels of *NtUCP1* appeared to recover to the normoxia level after 24h of hypoxia treatment (Supplementary Fig. S9C). Under hypoxic conditions, transcript levels of cytochrome oxidase (*COX*) and F1-FO ATP synthase were decreased in the P07 plants and increased when both P07 and WT plants were grown in normal oxygen concentration. However, the *COX* downregulation under hypoxia was lower in P07 plants compared with the change in WT plants.

Since the differential gene expression in the P07 plants under hypoxia was more pronounced after 8h treatment, this time point was used to analyse the metabolite and carbohydrate profiles in both genotypes. As expected, sucrose and lactate content were increased in both P07 and WT plants under hypoxic conditions (Fig. 5C). Although the sucrose content was similar, the lactate level was 2-fold higher in P07 compared with WT plants when grown in a normal oxygen concentration.

Hypoxia has a major effect on amino acid metabolism, but this varies among species, tissues, and developmental stages (Rocha et al, 2010; Narsai *et al.*, 2011). The alanine content increased 4-fold in WT plants grown in hypoxic conditions when compared with plants grown under normoxia (Fig. 5D). However, P07 plants had 3-fold higher alanine levels compared with the WT plants under normoxia. Glutamine, glycine, and serine were all increased in both P07 and WT plants under hypoxic conditions (Fig. 5D). Interestingly, the glycine content was not altered in P07 and WT plants grown under normoxia. Glycine has been shown to be the only metabolite significantly affected by UCP1 insertional knockout in *Arabidopsis* (Sweetlove *et al.*, 2006).

Intermediates of mitochondrial metabolism were also profiled. Pyruvate content did not change in WT plants grown under hypoxia for 8h, most likely because of its conversion to alanine. However, pyruvate was increased by over 2-fold in P07 plants under normoxic conditions. The most marked change was found for fumarate, which increased 6-fold in the WT plants subjected to hypoxia when compared with plants under normoxia (Fig. 5E). Interestingly, the fumarate level did not changed in P07 plants under hypoxia, but the fumarate content was already 5-fold higher in P07 compared with WT plants when these were grown at normoxia. The succinate content was increased by 2-fold in plants under hypoxic conditions and was also 2-fold higher in P07 plants under normoxia (Fig. 5E). Malate and citrate was also found to be slightly increased in P07 plants grown under both normoxic and hypoxic conditions (Fig. 5E).

**Fig. 6.**
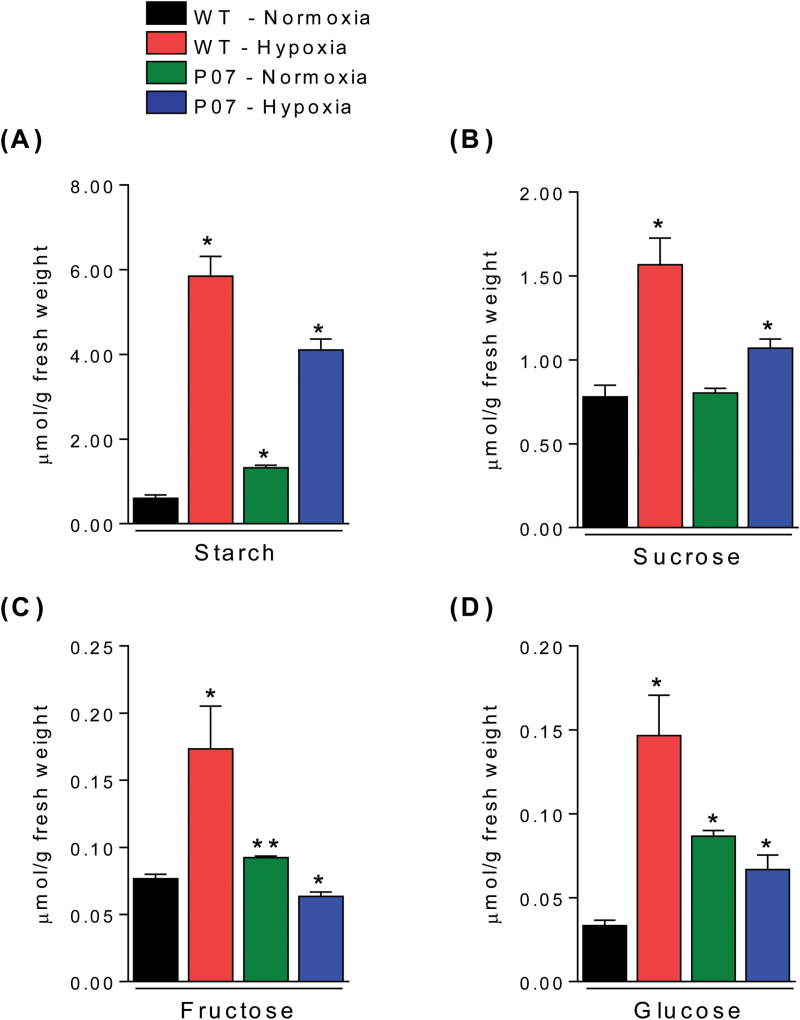
Absolute quantification of carbohydrate content in AtUCP1-overexpressing and WT plants grown for 8h under hypoxia. Insoluble carbohydrates in the form of starch (A) along with soluble carbohydrates in the form of sucrose (B), fructose (C), and glucose (D) were quantified in the aboveground parts of tobacco plants. Bars represent means±SEM. **P<0.05; *P<0.1 (significantly different from the WT under normoxia).

The effect of AtUCP1 overexpression on carbon fixation was assessed by the quantitation of starch and soluble sugar content in P07 and WT plants grown under hypoxia and normoxia. In the case of hypoxia, it is known that energy expenditure decreases due to impaired OXPHOS and that fermentable sugars tend to accumulate within the cells (Hossain and Uddin, 2011; Narsai *et al.*, 2011). It was found that the starch content increased 2-fold in P07 plants under normoxia (Fig. 6A). When subjected to hypoxic conditions, the starch content in P07 plants was increased by 3-fold, while WT plants showed a 10-fold increase. Sucrose (Fig. 6B) and fructose (Fig. 6C) content were not significantly different between P07 and WT plants grown under normoxia, but sucrose accumulated in both under hypoxic conditions, while fructose accumulated only in WT plants. The sucrose content was increased 1.25-fold in P07 plants and 2-fold in WT plants, while fructose was decreased 2-fold in P07 and increased 3-fold in WT plants under hypoxic conditions. Finally, the glucose content was found to be slightly decreased in P07 plants but increased 5-fold in WT plants if growth was performed under hypoxia (Fig. 6D). However, the glucose level was 2-fold higher in P07 plants compared with the WT when both were grown under normoxia.

## Discussion

UCP1 overexpression in plants has been shown to have a beneficial impact, mostly attributed to a reduction of ROS production and improvement of cellular redox homeostasis (Brandalise *et al.*, 2003; Smith *et al.*, 2004; Begcy *et al.*, 2011; Chen *et al.*, 2013). UCP1 appears to provide an escape valve to decrease ROS production under stress conditions in which ATP synthase is not capable of dissipating the electrochemical gradient across the mitochondrial inner membrane (Krauss *et al.*, 2005; Figueira *et al.*, 2013). Nevertheless, it has been clearly demonstrated that, under normal physiological conditions, ROS production and degradation within cells is tightly controlled by the inherent mitochondrial antioxidant machinery and does not reach harmful levels (Figueira *et al.*, 2013). Thus, it appears that the beneficial effects of UCP1 overexpression when plants are grown under normal conditions are most likely not a result of a decreased production of ROS. In this study, a new hypothesis is proposed for the beneficial effects caused by UCP1 overexpression in plants. Our data support the notion that a hypoxic response caused by the exacerbated AtUCP1 expression and consequential chronical unbalance between oxygen consumption and ATP synthesis is the main factor responsible for the beneficial effects observed in plants overexpressing this gene product. This stress condition imposed on the cell would trigger a reconfiguration of cellular energy metabolism.

Recently, it was found that AtUCP1 overexpression in tobacco resulted in decreased ATP synthesis, and this may trigger a signalling process that induces mitochondrial biogenesis resulting in potent antioxidant responses and therefore better adaptation of plants to broad stress conditions (Barreto *et al.*, 2014). Two important factors arose from our previous findings. The first was that the stress-responsive genes induced in AtUCP1-overexpressing plants are not strictly limited to the mitochondrial antioxidant machinery and therefore ATP reduction is not likely to be the sole cause of this broad adaptation. In addition, these previous findings showed alterations of mitochondrial morphology following AtUCP1 overexpression in plants. This revealed the presence of doughnut-shaped mitochondria, which in humans reflect a disruption of energy homeostasis when cells are subjected to hypoxia or when the chemical uncoupler carbonyl cyanide *p*-trifluoromethoxyphenylhydrazone was added to the culture media (Liu and Hajnóczky, 2011). Therefore, these misshapen mitochondria may be a result of the imbalance between ATP production and oxygen consumption, and not simply a reduction of ATP synthesis (Liu and Hajnóczky, 2011).

Transcriptome analysis of AtUCP1-overexpressing plants revealed upregulation of genes encoding proteins commonly induced by hypoxia, such as ACS, PDC, ADH, LDH, and GABA-T, which appears contradictory to the observed increase in mitochondrial biogenesis. Nevertheless, it is well established that increased mitochondrial biogenesis increases cellular oxygen consumption (O’Hagan *et al.*, 2008). The key factor here is that the compensatory effect on mitochondrial biogenesis-induced oxygen consumption does not overcome the problems of cells in meeting energy demands, since the gene encoding AtUCP1 is constitutively overexpressed. Thus, it is possible that the combination of increased mitochondrial biogenesis and AtUCP1 overexpression exacerbates the imbalance between oxygen consumption and ATP synthesis. It is known that hypoxic pathways are induced by this imbalance, rather than by decreasing oxygen concentration, without altering ATP production (Zabalza *et al.*, 2009).

In this study, the content of key metabolites and genes in P07 and WT plants grown under hypoxic and normoxic conditions were profiled with the aim of identifying specific markers of hypoxic adaptation. This showed that expression of the gene encoding the endogenous NtUCP1 was downregulated 10-fold in both P07 and WT plants exposed to short-term hypoxia. These results support the concept that, in order to prevent excess oxygen consumption under hypoxic conditions, the NtUCP1-encoding gene is strongly repressed. Lactate and alanine were among the most accumulated metabolites. While lactate is a by-product of fermentative glycolysis, alanine is involved in preventing the entry of pyruvate into mitochondria (Rocha *et al.*, 2010; Narsai *et al.*, 2011). The conversion of pyruvate into alanine can be performed by AlaAT or GABA-T, which appear to be partially redundant pathways (Rocha *et al.*, 2010). Interestingly, GABA-T was upregulated and AlaAT was repressed in P07 compared with WT plants. Under hypoxic conditions, the two pathways were induced in both P07 and WT plants. The most significant alterations to TCA intermediates were the 2- and 4-fold increases in succinate and fumarate content, respectively, in P07 plants grown under normoxic conditions. Fumarate was also increased in plants grown under hypoxic conditions in both genotypes. In human tissues, fumarate and succinate that accumulate in the mitochondria leak to the cytosol and inhibit a family of prolyl hydroxylase enzymes. These enzymes are responsible for tagging the hypoxia inducible factor-1 (HIF-1) complex, the master regulator of hypoxic response in mammals, for degradation. As a result, there is an enhancement of glycolysis and cells become more resistant to apoptotic signals (King *et al.*, 2006; Koivunen *et al.*, 2007). Since the prolyl hydroxylase enzymes have orthologues in the *Arabidopsis* genome (Vigani *et al.*, 2013), it would be worth investigating whether succinate and especially fumarate accumulation has the same consequence in plants. Activation of the hypoxic pathways was then investigated by identifying differentially expressed TFs. Of the 18 most differentially expressed TFs, 13 were regulated by the hypoxic conditions. In addition, the group of genes co-expressed with these TFs was also enriched in transcripts related to fermentation and hypoxic adaptation.

One remaining question is the beneficial impact of AtUCP1 overexpression on carbon assimilation and stomatal conductance. Previous findings showed that an insertional knockout of UCP1 in *A. thaliana* resulted in impaired photorespiration (Sweetlove *et al.*, 2006). The improvement in carbon assimilation in UCP1-overexpressors seems not to be the result of any improvement in photorespiration, since the transcripts mapped to this pathway are slightly downregulated and glycine levels are not altered. Nevertheless, it has been observed that TCA intermediates accumulate and function as CO_2_ sensors inside the cell and regulate photosynthesis (Hedrich and Marten, 1993). An extensive study performed on different lines of succinate dehydrogenase antisense tomato plants showed that these plants accumulated succinate, resulting in altered concentrations of organic acids in guard cells and increased stomatal conductance (Araujo *et al.*, 2011; Fuentes *et al.*, 2011). On the other hand, accumulation of fumarate in fumarase antisense tomato plants displayed the exact opposite effect, i.e. decreased stomatal opening and photosynthesis (Nunes-Nesi *et al.*, 2007; Cavalcanti *et al.*, 2014). It is not clear how the accumulation of both of these TCA intermediates would improve photosynthesis in UCP1-overexpressing plants. It is hypothesized that if these organic acid molecules act as sensors for hypoxic conditions, this would lead to an increase in stomatal conductance and gas exchange as a means of counteracting decreased oxygen concentrations. In turn, this would lead to increased uptake of CO_2_ into chloroplasts and subsequent stimulation of carbon assimilation. Increased carbon assimilation is necessary for increasing carbon sinks to compensate for the more ineffective ATP synthesis through fermentative glycolysis (Hossain and Uddin, 2011; Narsai *et al.*, 2011). The results of this study indicate an increase in carbon fixation by P07 plants, as deduced from the increased starch and glucose content when plants were grown under normoxia. It is known that increased carbohydrate content (e.g. glucose) can have a negative impact on the expression of photosynthesis-related genes in plants (Paul and Foyer, 2001). It is suggested suggest that the downregulation of genes encoding PSI, PSII, and chlorophyll-synthesis transcripts may be the result of a feedback mechanism imposed by the increased carbohydrate levels. It was also demonstrated that, under hypoxic conditions, the P07 plants are still capable of expending energy, accumulating fewer carbon sinks in the form of glucose and fructose compared with the WT plants. This suggests that AtUCP1 overexpression confers better adaptation of plants to decreased oxygen concentrations. It is still not clear whether these alterations in transcript levels lead to corresponding changes in the content of the encoded proteins of the photosystems, but they do not appear to negatively affect the physiological parameters of photosynthesis or the chlorophyll content.

Taken together, the data presented in this work have unravelled a complete reconfiguration of energy metabolism in response to the increased uncoupling activity. In this scenario, plants overexpressing AtUCP1 also meet their energy demands by fermentative glycolysis due to an imbalance of ATP production and oxygen consumption in the mitochondria. The increased photosynthetic carbon assimilation and gas exchange are a consequence of the hypoxic adaptation. As plants with a constitutive activated hypoxic pathway perform better under several abiotic stresses (Gibbs *et al.*, 2011), the performance of UCP1 transgenic plants under an array of biotic and abiotic stresses may also arise from this metabolic reconfiguration.

## Supplementary data

Supplementary data are available at *JXB* online.

Supplementary Fig. S1. Pipeline for accessing P07 transcriptome in relation to its WT counterpart as described in Material and methods.

Supplementary Fig. S2. Pipeline for transcription factor analysis using Genevestigator.

Supplementary Fig. S3. qRT-PCR validation of the transcriptome.

Supplementary Fig. S4. Distribution DEGs among COG classifications.

Supplementary Fig. S5. MapMan overview of the transcripts related to stress response.

Supplementary Fig. S6. MapMan overview of the transcripts related to nucleotide synthesis pathways.

Supplementary Fig. S7. MapMan overview of the transcripts related to starch and sucrose metabolism.

Supplementary Fig. S8. MapMan overview of the transcripts related to glycolysis, the tricarboxylic acid cycle, and mitochondrial electron transport chain.

Supplementary Fig. S9. qRT-PCR for plants submitted to hypoxic conditions.

Supplementary Table S1. (A) Output of *N. tabacum* WT and P07 plants transcriptome sequencing. (B) Distribution of RNA-seq-generated contigs, and number of annotated contigs and differentially expressed genes.

Supplementary Table S2. (A) List of upregulated genes in transgenic plants overexpressing UCP1 (P07) when compared with WT. (B) List of downregulated genes in transgenic plants overexpressing UCP1 (P07) when compared with WT.

Supplementary Table S3. (A) Genes selected for experimental expression validation and the corresponding primers used for qRT-PCRs. (B) Genes selected for evaluation of the hypoxic response and the corresponding primers used for qRT-PCs.

Supplementary Table S4. Gene Ontology (GO) assignments of the differentially expressed genes in UCP1-overexpressing plants when compared with its WT counterpart.

Supplementary Table S5. (A) KEGG mapping of the upregulated DEGs in UCP1-overexpressing plants when compared with its WT counterpart. (B) KEGG mapping of the downregulated DEGs in UCP1-overexpressing plants when compared with its WT counterpart.

Supplementary Table S6. List of differentially expressed chloroplastic genes shown graphically in Fig. 2.

Supplementary Table S7. MapMan enrichment analysis of genes co-expressed with differentially expressed transcription factors.

Supplementary Data

## Abbreviations:

COG: Clusters of Orthologous Groups
contig: contiguous sequence
DEG: differentially expressed genes
FDR: false discovery rate
GO: Gene Ontology
KEGG: Kyoto Encyclopedia of Genes and Genomes
NMR: nuclear magnetic resonance
OXPHOS: oxidative phosphorylation
PS: photosystem
qRT-PCR: quantitative reverse transcription PCR
ROS: reactive oxygen species
RPKM: reads per kb per million
TCA: tricarboxylic acid cycle
TF: transcription factor
UCP: mitochondrial uncoupling protein
WT: wild type.
