# Supplementary material for: Overexpression of mitochondrial uncoupling protein 1 (UCP1) induces a hypoxic response in Nicotiana tabacum leaves
Source: J Exp Bot. 2015 Oct 22;67(1):301–13. doi: 10.1093/jxb/erv460 (PMC4682437; doi:10.1093/jxb/erv460)
Supplement: Supplementary Data [file supp_67_1_301__index.html]

Overexpression of mitochondrial uncoupling protein 1 (UCP1) induces a hypoxic response in Nicotiana tabacum leaves — Overexpression of mitochondrial uncoupling protein 1 (UCP1) induces a hypoxic response in Nicotiana tabacum leaves — Supplementary Data 

# Overexpression of mitochondrial uncoupling protein 1 (UCP1) induces a hypoxic response in *Nicotiana tabacum* leaves

## Supplementary Data

Data files

- Supplementary\_FigS1\_S9.pdf - Supplementary Data
- Supplementary\_table2.xlsx - Supplementary Data
- Supplementary\_table1\_table3\_table7.xlsx - Supplementary Data
