# Supplementary material for: Overexpression of mitochondrial uncoupling protein 1 (UCP1) induces a hypoxic response in Nicotiana tabacum leaves
Source: J Exp Bot. 2015 Oct 22;67(1):301–13. doi: 10.1093/jxb/erv460 (PMC4682437; doi:10.1093/jxb/erv460)

Journal of Experimental Botany

SUPPLEMENTARY DATA

Overexpression of mitochondrial uncoupling protein 1 (UCP1) induces a hypoxic response in Nicotiana tabacum leaves

Pedro Barreto, Vagner Okura, Izabella A. Pena, Renato Maia, Ivan G. Maia and Paulo Arruda

Supplementary Figure S1. Pipeline for accessing P07 transcriptome in relation to its WT counterpart as described in Material and Methods.

Total RNA was isolated from wild type (WT) and transgenic plants overexpressing UCP1 (P07) for single-end RNAseq libraries preparation and sequencing. The raw reads were submitted to quality control and filtering steps, digitally normalized and assembled into contigs. These contigs were mapped into *S. lycopersicum* proteome. For gene expression amounts quantification (RPKM), the reads that were submitted to quality control and filtering was mapped into the assembled contigs.

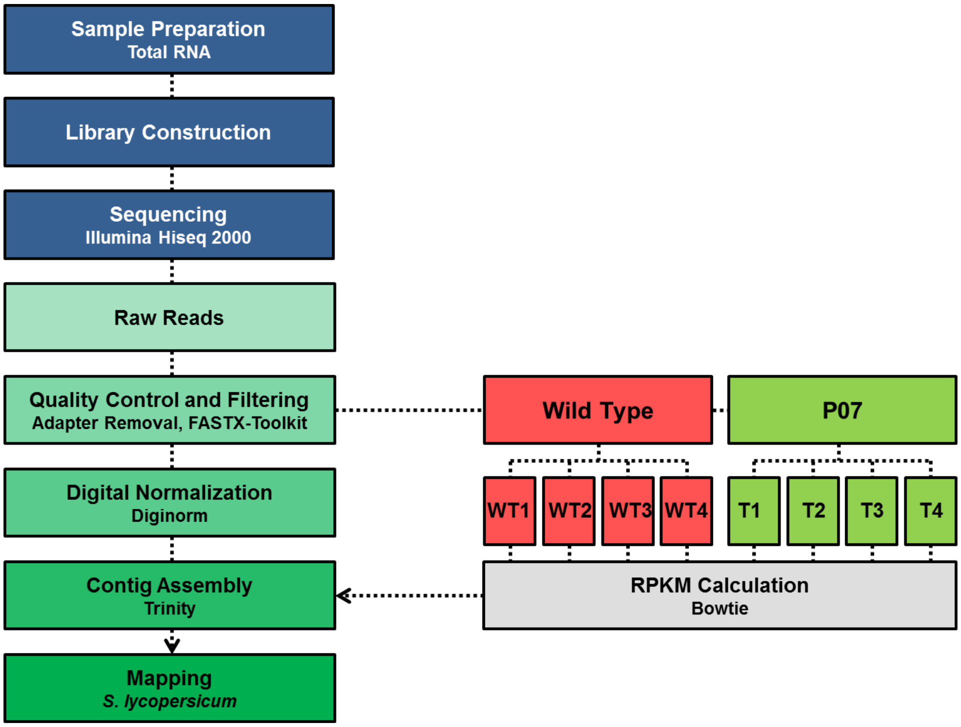

Supplementary Figure S2. Pipeline for transcription factor analysis using Genevestigator

The top 9 up- and down-regulated transcription factors (TF) were selected among the 283 identified in the transcriptome. Datasets (DS) containing experiments that significantly affect each transcription factor expression by more then 2-fold (P-value < 0.01) were selected. We select the 100 most coexpressed genes (with a Pearson correlation > 0.5) that are negatively (CE, red boxes) and positively correlated (CE, green boxes) with each transcription factor and pooled them in 4 different groups. Positive and negative correlated with the up-regulated TFs and positive and negative correlated with the down-regulated TFs. An enrichment analysis were performed using the whole Arabidopsis genome as background.

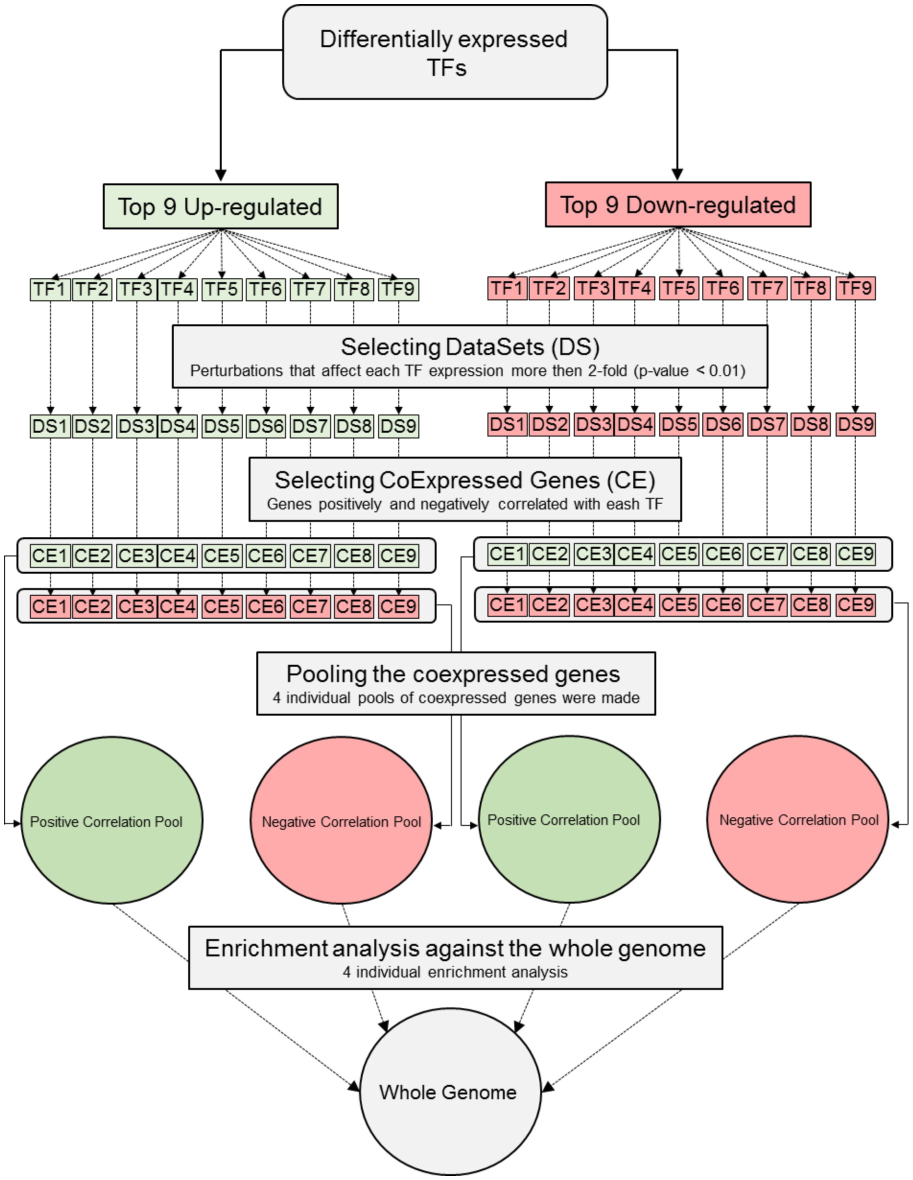

Supplementary Figure S3. qRT-PCR validation of the transcriptome

(A) Thirteen transcripts were selected from the transcriptome for qRT-PCR validation using the same RNA as for RNA-seq. Actin1 has been used as an internal reference (B) Correlation of gene expression ratio between RNA-seq data and qRT-PCR data resulted in an correlation value (r^2) of 0.9693. Data are plotted as Log_2 of fold-values. Bars represent mean ± SEM for four biological replicates.

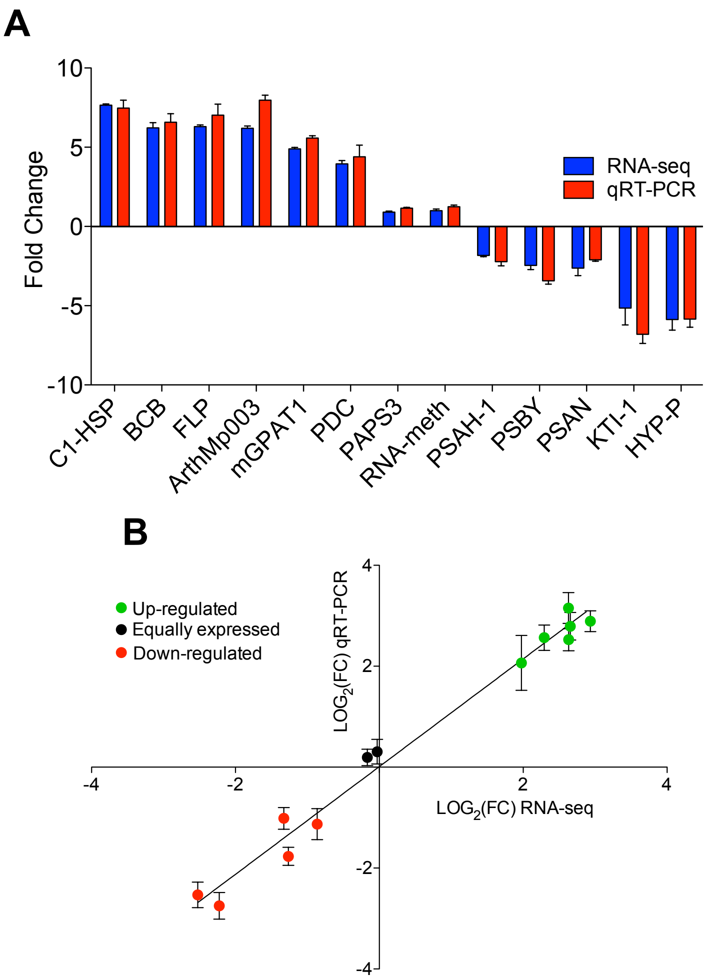

Supplementary Figure S4. Distribution DEGs among COG classifications.

DEG sequences used as queries against the COG database yielded 1,679 classifications of up-regulated genes (green bars) and 550 classifications of down-regulated genes (red bars) in *At*UCP1 over-expressing plants compared to WT plants.

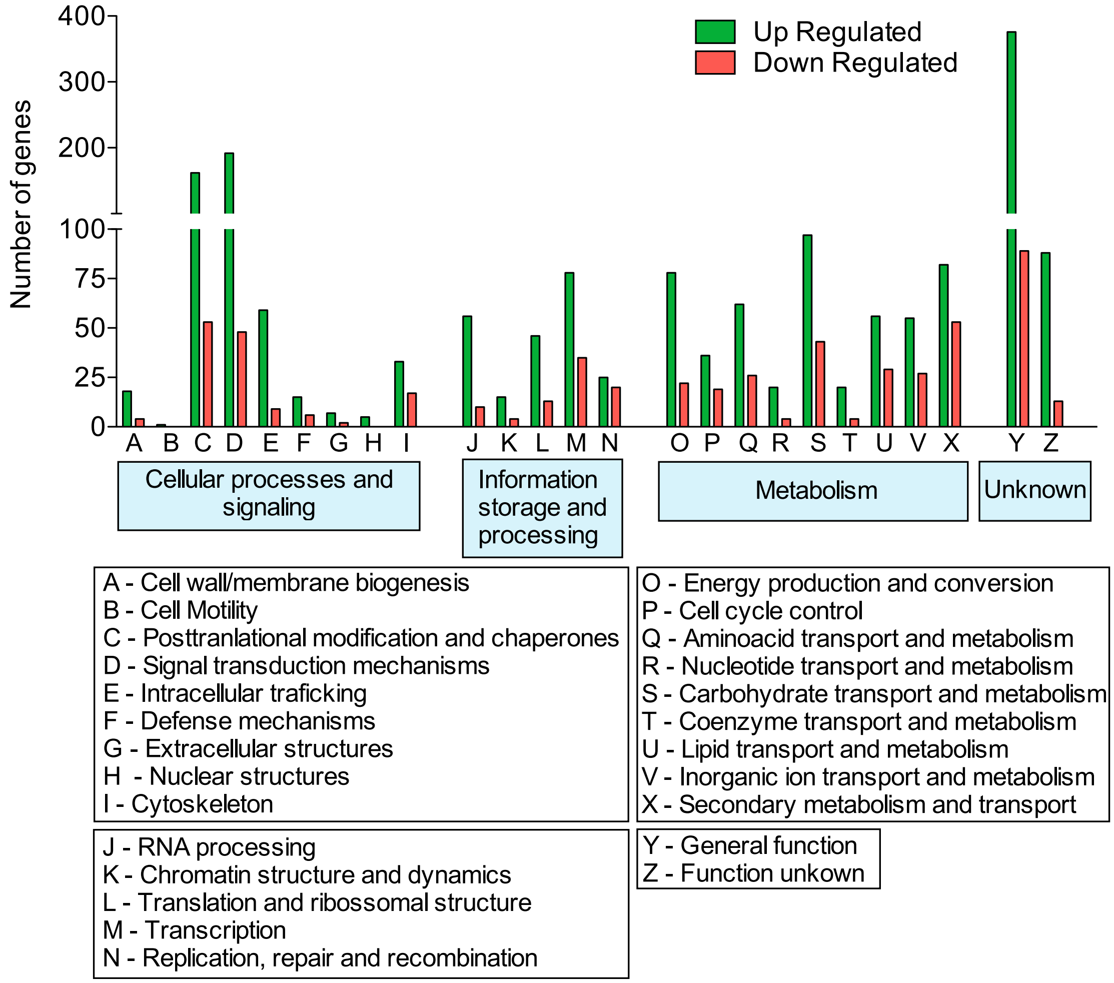

Supplementary Figure S5. MapMan overview of the transcripts related to stress response.

A broad induction of biotic and abiotic stress responsive genes is observed. Log_2 ratio of the transgenic to the WT for individual genes was plotted onto boxes. Green boxes indicate up-regulated genes and red boxes indicate down-regulated genes.

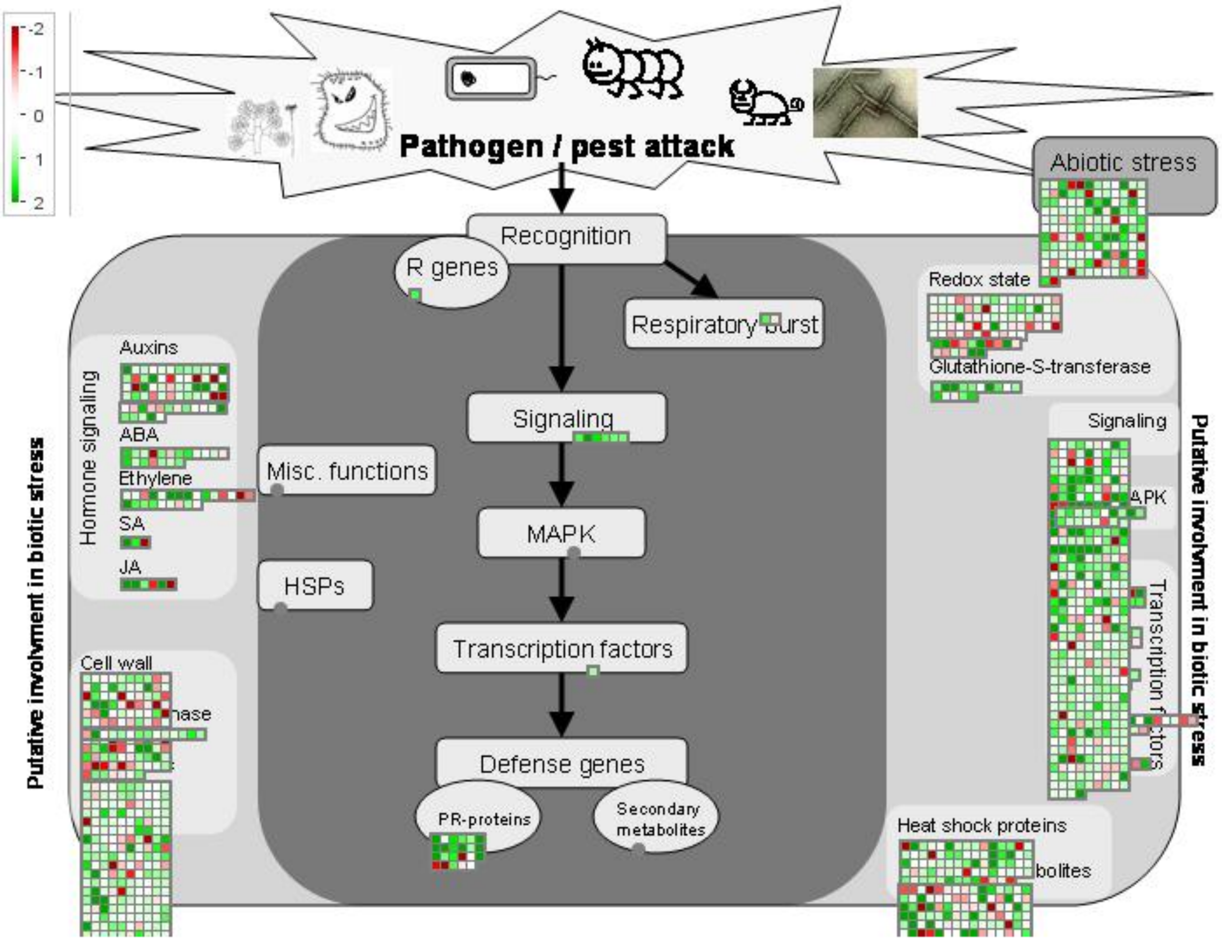

Supplementary Figure S6. MapMan overview of the transcripts related to nucleotide synthesis pathways.

We observe a 3-fold up-regulation in Guanylate Kinase transcript, whose gene product converts GMP into GTP, a classic inhibitor of UCPs. Log_2 ratio of the transgenic to the WT for individual genes was plotted onto boxes. Green boxes indicate up-regulated genes and red boxes indicate down-regulated genes.

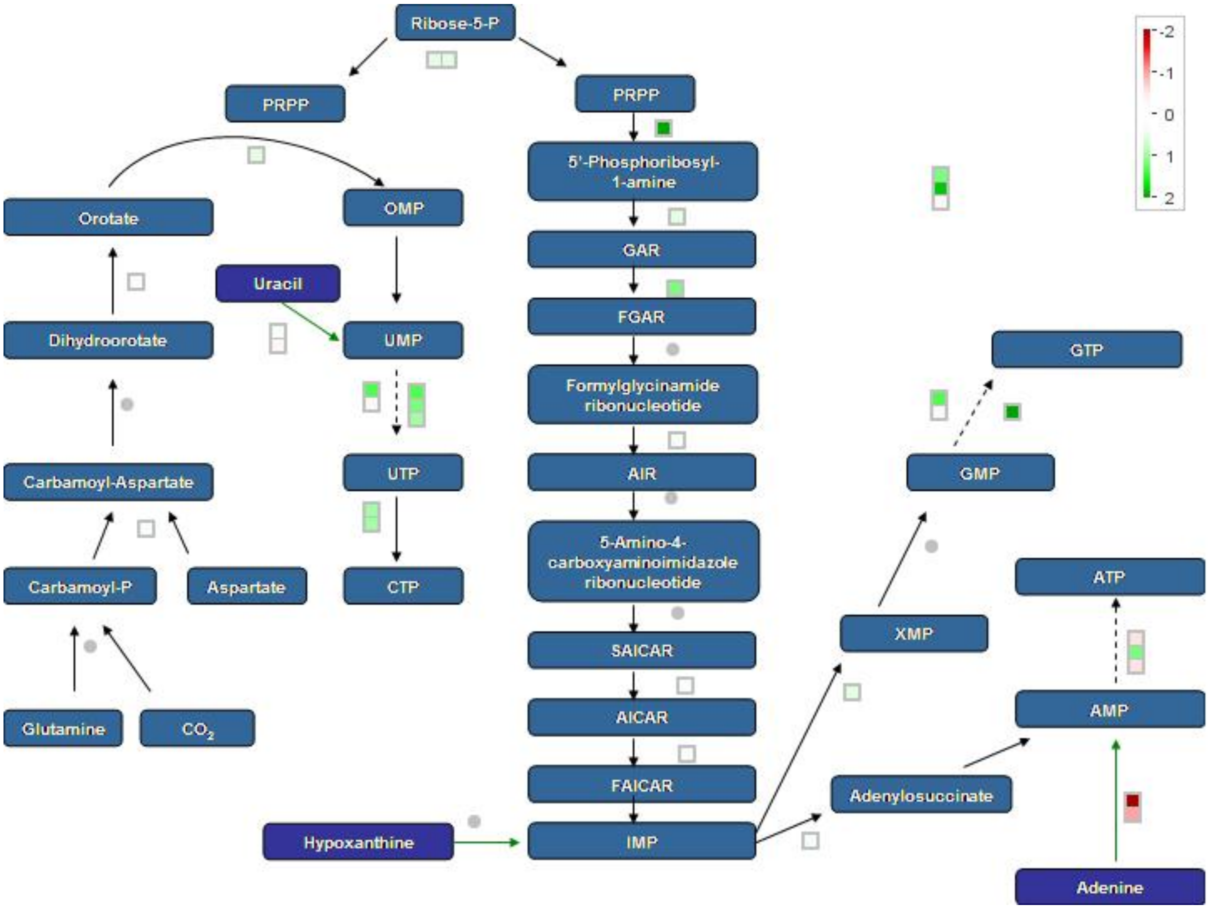

Supplementary Figure S7. MapMan overview of the transcripts related to starch and sucrose metabolism.

(A) Transcripts mapped into sucrose metabolism. (B) Transcript mapped into starch metabolism. Log_2 ratio of the transgenic to the WT for individual genes was plotted onto boxes. Green boxes indicate up-regulated genes and red boxes indicate down-regulated genes.

A B

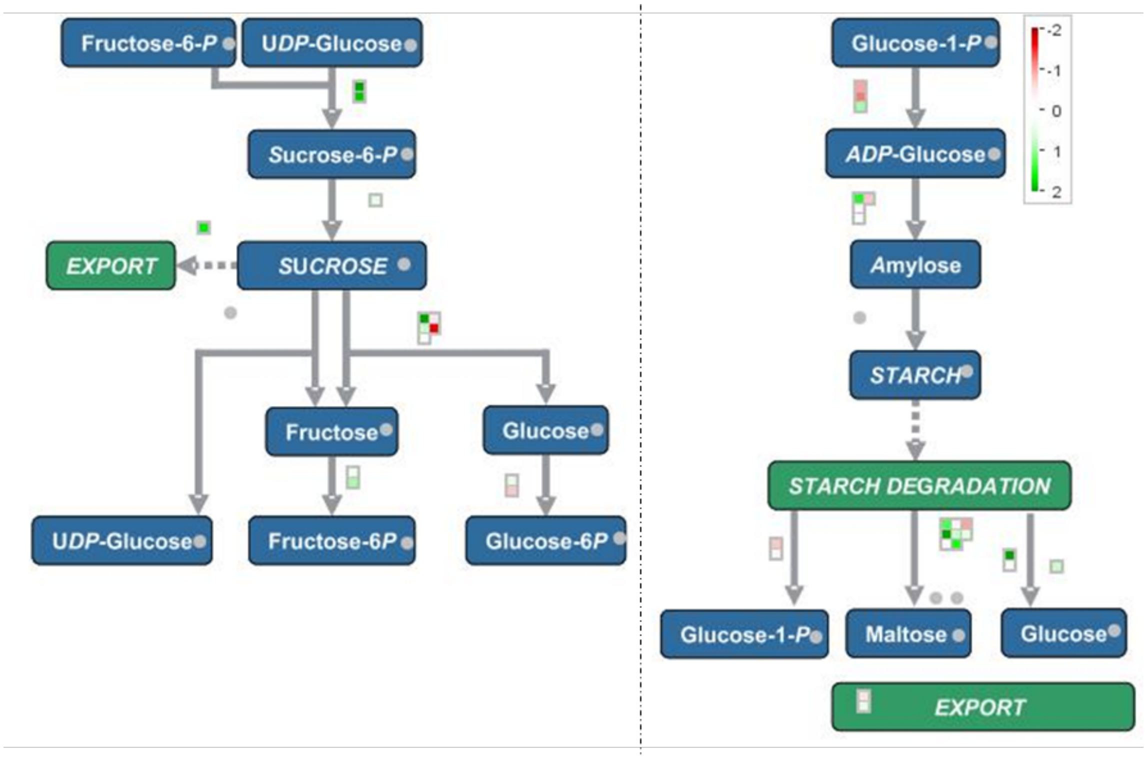

Supplementary Figure S8. MapMan overview of the transcripts related to glycolysis, tricarboxylic acid cycle and mitochondrial electron transport chain.

Transcripts mapped into the initial step of glycolysis, fermentation and electron transport chain are up-regulated in transgenic plants. Interestingly, gene products that are related to the remove of intermediates from tricarboxylic acid cycle, such as ATP citrate lyase and malate dehydrogenase are up-regulated in transgenic plants. Log_2 ratio of the transgenic to the WT for individual genes was plotted onto boxes. Green boxes indicate up-regulated genes and red boxes indicate down-regulated genes.

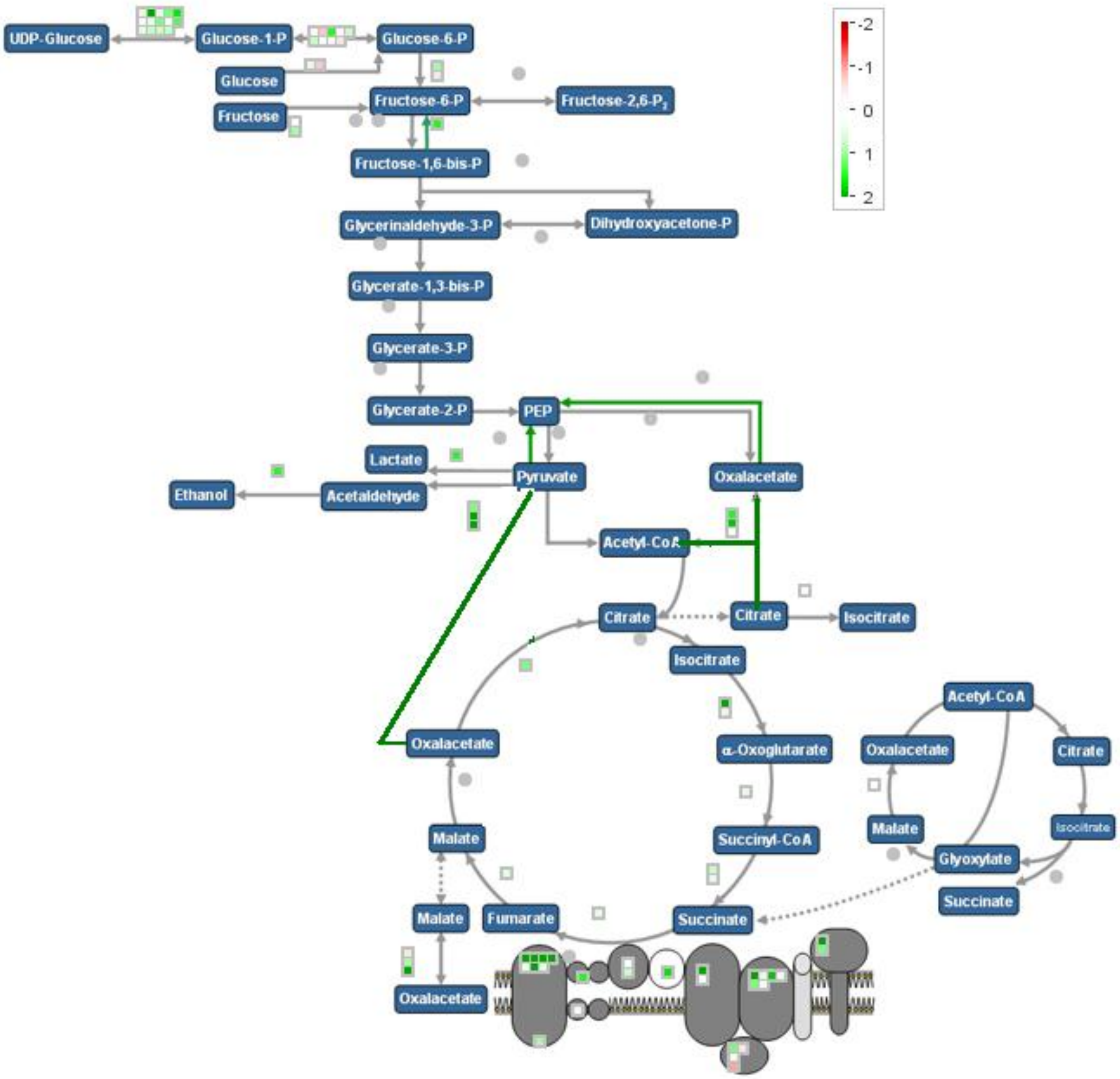

Supplementary Figure S9. qRT-PCR for plants submitted to hypoxic conditions

Samples for the plants exposed to hypoxic conditions were collected at 0, 2, 8, 24 and 48 hours of exposure to a 5% O_2 atmosphere and after 8 hours of recovery under normoxic conditions. Relative quantification data were presented for (A) ADH, (B) RAP2.2, (C) the endogenous *Nicotiana tabacum* UCP1 and (D) ATPS. Bars represent mean ± SEM of four biological replicates. Each biological replicate contains a pool of the aboveground parts of three plants. Bars with * (P < 0.05) and ** (P < 0.1) differ significantly from the WT.

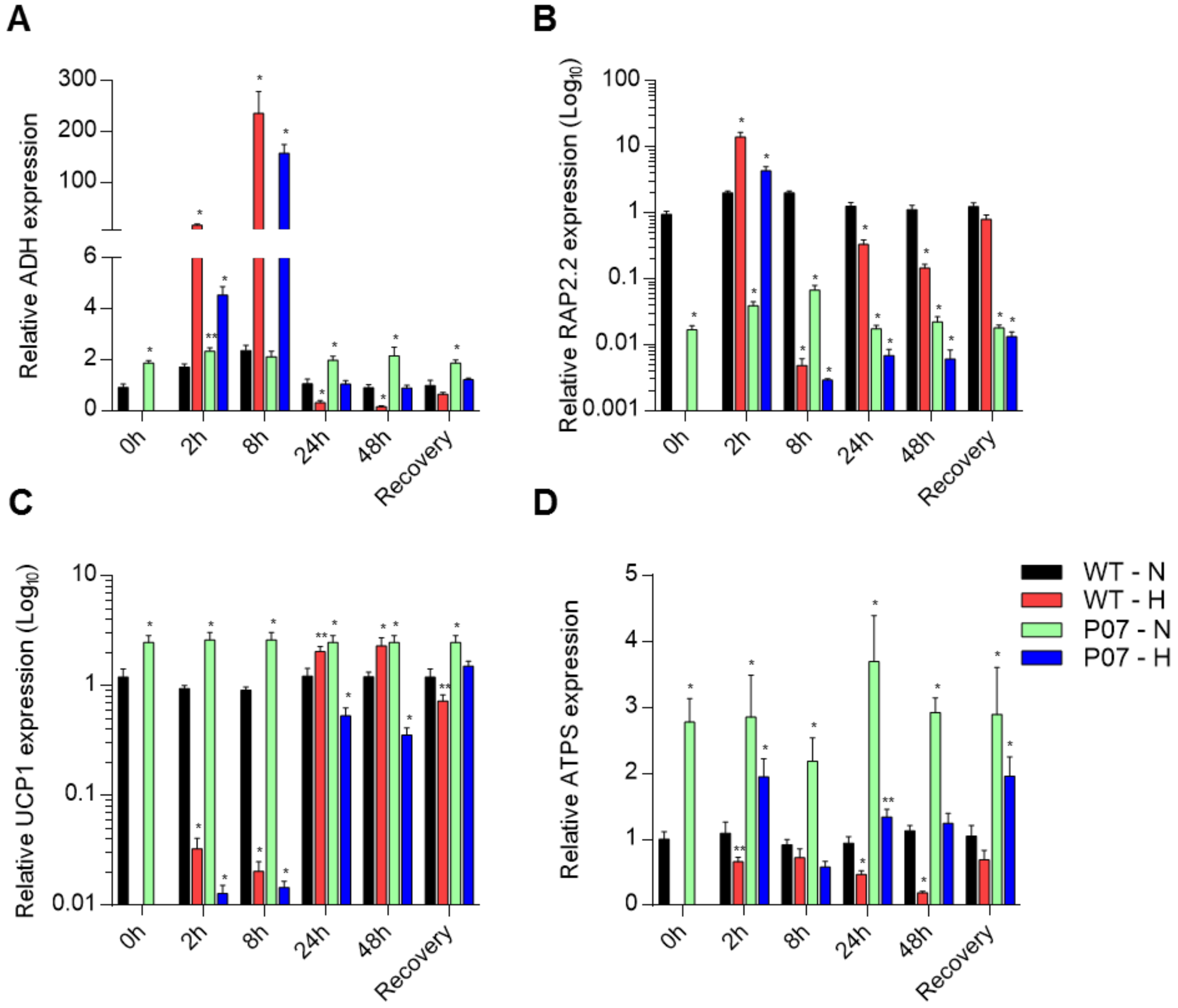

Supplement: Supplementary Data [file supp_erv460_Supplementary_FigS1_S9.pdf]
